# Anastomosis Button

**Published:** 1893-07

**Authors:** 


### ANASTOMOSIS BUTTON.

This device introduced by Dr. J. B. Murphy, of Chicago for uniting different structures has now been tested in a sufficient number of cases to afford a reasonable reliance upon its efficacy. I had an opportunity during the meeting of the American Medical Association at Milwaukee to witness its experimental application in dogs at the county hospital by Drs. Murphy, Ruth and Bacon. It was introduced for the union of the gall-bladder and duodenum, the stomach and parites, end to end juncture of the of the intestinal canal after resection, and the lower portion of the rectum with the sigmoid flexure of the colon, illustrating the treatment of stricture of the rectum. The results were entirely satisfactory in the demonstration of the adaptation of the button for anastomosis, and there can remain but little doubt of a communication with surrounding adhesion being accomplished by it, with more expedition than by processes heretofore adopted.

A number of successful operations with this button have been reported by Dr. Murphy, and it has been used by Dr. Walker, of Detroit, Dr. Ruth, of Keokuk, and others with satisfactory results.

A modification of the anastomosis button has been suggested by Dr. Richardson, of Boston, in the attachment of a cylinder, some two inches long, to one limb of the button, which serves as a drainage tube from the cavity into which the other portion of the button is inserted. It was my good fortune to witness the application of this by Dr. Murphy at St. Joseph's Hospital in Chicago, in a case of gall stones with the walls of the gall-bladder so much contracted and so attached by adhesions that they could not be brought in contact with the duodenum or with the external points of the abdomen. This obviates the inconvenience of tamponing around an ordinary drainage tube to prevent the escape of exudation from the gall-bladder into the peritoneal cavity and meets the requirements completely, until the tissues are agglutinated around the tract of the cylinder.

The greatest obstacle to the general adoption of the anastomosis button is the liability of this solid body sloughing through the tissues where there is much tension, and escaping into the peritoneal cavity, or on the other hand, when it is detached by atrophic desintegration of the included edges of the viscus into the small intestines, that it may be arrested in the course of this canal or at the ileo-cœcal valve. Thus far nothing of this kind has occurred, and it is not likely to take place in the attachment of the small and large intestine, when the caliber of bowel can present no impediment to the exit of the button by the rectum.

I am impressed with the advantages to be gained by this mode of anastomosis, and consider that the employment of a smaller button than either of those used heretofore, may be well adopted for cholecysto-duodenal anastomosis and thus obviate all possible trouble in the intestine canal.

J. M'F. G.

